
JOURNAL INFORMATION
==============================
NLM Title Abbreviation: ScientificWorldJournal
Journal ID: ScientificWorldJournal
NLM Journal ID: 101131163
Journal ID: TSWJ

The Scientific World Journal

ISSN: 2356-6140
EISSN: 1537-744X
Publisher: Wiley

ARTICLE INFORMATION
==============================
PMCID: PMC5956433
PMID: 15349565
DOI: 10.1100/tsw.2004.84
Article ID: 953563
Article version: 1
Subjects: Original Article

Urologic Problems in Patients with Acquired Immunodeficiency Syndrome

Staiman Victoria R.
Lowe Franklin C.
St. Luke's-Roosevelt Hospital Center, Columbia College of Physicians and Surgeons, USA
Academic Editor: Anthony Atala

Publication date: 2004
Electronic publication date: 2004 Jun 28
Volume: 4
First page: 427
Last page: 437
Copyright: Copyright © 2004 Victoria R. Staiman and Franklin C. Lowe.
Copyright year: 2004
License: This is an open access article distributed under the Creative Commons Attribution License, which permits unrestricted use, distribution, and reproduction in any medium, provided the original work is properly cited.
License URL: https://creativecommons.org/licenses/by/3.0/

==============================
Original Articles
*TheScientificWorldJOURNAL* (2004) 4 (S1), 427–437
ISSN 1537-744X; DOI 10.1100/tsw.2004.84

### Urologic Problems in Patients with Acquired Immunodeficiency Syndrome

Victoria R. Staiman, MD[1] and Franklin C. Lowe, MD, MPH[2]

*[1]Senior Resident in Urology, Squier Urologic Clinic and [2]Associate Director of Urology, St. Luke's-Roosevelt Hospital Center, Clinical Associate Professor of Urology, Columbia College of Physicians and Surgeons*

**E-mails**: vstaiman@hotmail.com; Lowe21@aol.com

*Previously published in the Digital Urology Journal*

**DOMAIN**: urology

Approximately 600,000 cases and 360,000 deaths related to AIDS have been reported to the Centers for Disease Control in the United States.[1] However, from January 1994 to June 1996, the incidence of AIDS has finally stabilized, with approximately 15,000 cases per quarter reported.[1] While AIDS appears to involve all organ systems, the genitourinary system is both primarily and secondarily affected. This article reviews the genitourinary manifestations of the human immmunodeficiency virus (HIV) and the most current treatment options available.

### HIV TRANSMISSION

HIV particles have been isolated from blood, plasma, tears, urine, saliva, breast milk, as well as seminal fluid and pre-ejaculate fluid and cervical and vaginal secretions.[2,3] The concentration of viral particles has been found to be greater in the semen than in the vaginal secretions. In addition, the concentration of virus in the blood is relatively low, and replicates slower in asymptomatic infected patients, while the concentration is greater and replicates more rapidly during the later stage of the disease.[4,5] The transmission of HIV; therefore, can occur through blood and blood products, vertically from mother to child during childbirth, or during intimate sexual contact.[2,6] Sexual contact accounts for 75 to 85% of the 28 million worldwide cases of HIV-infection to date.[7] Male to female transmission is approximately two times more common than female to male transmission.[4,8] The cervix is the most likely route of transmission in the female. Even though the vaginal epithelium is generally a barrier for infectivity, transmission can still occur by this route if ulcerations are present.

There has been some controversy as to whether male circumcision may have protective qualities from HIV transmission. From a worldwide analysis, there is a statistically significant association between the presence of a foreskin and the risk for HIV infection.[9] These studies report that an uncircumcised male has a 1.5 to 8.4 time risk of being infected with HIV than if circumcised. The foreskin provides a protected environment and large surface area for prolonged exposure between the genital epithelium and the infected genital secretions.[10] The uncircumcised penis lack the thick stratum corneum layer which develops on the glans after circumcision.[11,12] This layer is protective against abrasion and subsequently

HIV infectivity. In addition, the foreskin is associated with high numbers of lymphocytes and monocyte-macrophages. These cells are the targets for the HIV virus.[11,12] Finally, the uncircumcised male is at higher risk of acquiring other sexually transmitted diseases. These can increase the likelihood of HIV transmission.[7,13]

The commonly prescribed antiretroviral therapy appears to change the concentration of HIV-ribonucleic acid in both the blood and seminal fluid. This therapy may reduce the spread of HIV.[14]

### INFECTIONS OF THE URINARY TRACT

While the genitourinary system is both a primary site of HIV infection as well as a site for its complications, the genitourinary tract is generally protected. According to Miles and associates, urinary tract infections occur in 17% of patients, while there was a 16% incidence of urologic related symptoms.[15] Symptoms of urinary tract infections include dysuria, frequency, urgency, and hematuria, but many patients are relatively aymptomatic. *Escherichia coli*, which accounts for up to 80% of urinary tract infections in the general population, only accounts for 25% of urinary tract infections in HIV-positive patients. *Pseudomonas aeruginosa* is found in up to 33%.[16,17] Bacterial infections are more common in AIDS patients with CD4 counts of less than 200/ul. [18-21] In patients with low CD4 counts, neurological symptoms may also occur. Bladder areflexia and hyporeflexia is a common neurologic complication, which leads to urinary stasis, and ultimately infection.[22,23]

Although infection may be present, cultures are often negative. Most AIDS patients take prophylactic antimicrobials for opportunistic infections causing pneumonia or diarrhea and this renders culture negativity. Non-bacterial urinary tract infections (yeast, fungal, viral) are also common in this patient population, especially in patients with low CD4 counts, and negative cultures should alert the urologist to perform special cultures and stains.[18] Urine cultures obtained after prostatic examination or massage are more likely to be positive as the prostate gland is capable of harboring infectious agents. Therefore, HIV-infected patients should not just be treated with broad spectrum antibiotics for symptoms and suspected urine infection, but rather should be treated with antibiotics that are culture-specific.[18]

Hematuria is often found on urinalysis in many patients with HIV disease who are asymptomatic. In one study, hematuria was found in 25%, but was rarely found to be clinically significant.[24] Thus, asymptomatic HIV-positive patients with microhematuria do not require urologic evaluation unless previous urologic history or abnormal renal function is present.

### Prostatitis

Prostatitis is a result of urinary stasis due to either a dysfunctional or obstructed urinary system. The prostate gland usually has its own mechanism of defense for resisting bacterial infections (including spermine, spermidine, and prostatic antibacterial factor),[25,26] but in the HIV-infected patient, it is suspected that local immunodeficiency of the prostate fluid allows bacterial invasion.

The incidence of acute bacterial prostatitis is 1 to 2% in the general population, while it is 3% in asymptomatic HIV-infected patients and 14% in patients with AIDS.[27] The diagnosis of prostatitis is made by history of symptoms, digital rectal exam and by urine culture results. Again, *E. coli* is the typical causative agent in the general population, but HIV-infected patients are affected by both typical and atypical bacteria as well as viral and fungal agents. Other bacterial organisms involved in prostatitis include: *Staphylococcus aureus*, *Klebsiella pneumoniae*, *P. aeruginosa*, *Serratia marcescens*, and *Salmonella typhi*. *S. aureus* is often iatrogenically obtained and spread hematogenously[25,28], while the gram-negative organisms cause prostatitis via reflux of infected urine into prostatic ducts[25]. *Mycobacterium tuberculosis* and *Mycobacterium avium intracellulare*, which are responsible for pulmonary complications in many patients with AIDS, have also been recognized as a cause of prostatitis. In addition, viral agents such as HIV itself and cytomegalovirus (CMV) have been identified. CMV is extremely difficult to treat since it is found intracellularly in the epithelial layer of the genitourinary tract. *Cryptococcus neoformans* and *Histoplasma capsulatum* are fungal organisms reported to cause prostatitis in this patient population.[29,30] Due to the ability of the prostate to harbor organisms and the poor penetrance of antimicrobials to prostate tissue, HIV-infected patients often have persistent subclinical foci causing relapsing prostatitis.[31]

### Prostatic Abscess

Typically, prostatic abscesses are found in patients with pre-disposing factors such as diabetes mellitus, previous bladder catheterizations or instrumentations, or urinary obstruction; but, prostatic abscesses are clearly an emerging problem in the AIDS population.[32,33] During the first 12 years of the AIDS epidemic at our institution, only 1 HIV-positive patient had a prostatic abscess, while in the 2 subsequent years, 9 patients were diagnosed.[32-34] The most common symptoms include acute urinary retention, fever, dysuria, frequency, and perineal pain.[25] Clinical signs include and enlarged prostate in 75% of patients with prostatic tenderness and fluctuant mass noted in 35% and 16% respectively. Urine cultures are usually negative. In fact, in the 10 patients at our institution with prostatic abscesses, only 1 patient had an positive initial urine culture.[33] Eight patients underwent transurethral unroofing and 62% had positive operative cultures. Typical and atypical bacteria, as well as fungal agents were found, and it is; therefore, necessary for the urologist to consider atypical organisms otherwise the diagnosis may be missed. (Table 1)

The most common bacterial agents causing prostatic abscesses in HIV-infected patients are the Enterobacteriaceae and other gram-negative organisms. Two cases of mycobacterial prostatic abscesses were also recently reported.[33] Initial urinalysis and urine cultures will often show sterile pyuria.

Fungal abscesses (*H. capsulatum, Aspergillus*) have all been documented in AIDS patients and are usually secondary manifestations of primary infections.[33, 35-39] Therapy includes long-term, anti-fungal therapy; however, this treatment is not always capable of sterilizing prostatic foci, resulting in relapse.

Transrectal ultrasound is the most sensitive method of diagnosis.[34] Because antimicrobials penetrate prostatic tissue poorly, transurethral unroofing is also required. Long-term therapy is essential to avoid recurrent infection.

### VOIDING DYSFUNCTION

There is some controversy as to the degree of urinary symptoms suffered by patients with HIV. Gyrtrup and colleagues, in a prospective study, concluded that symptoms are fairly modest and that neurological bladder dysfunction only occurs rarely in the late stage AIDS patient.[40] Most urologists; however, have encountered patients with voiding dysfunction. In fact, it has also been reported that 16% of AIDS patients present with urologic manifestations (hematuria, dysuria, decreased force of stream, and acute urinary retention) and that neurological complications are the most frequent component causing urinary dysfunction.[41] Neurogenic voiding dysfunction portends a poor prognosis.[42] Urgency and incontinence are often secondary to bladder hyperreflexia caused by and upper motor neuron injury, either from encephalopathy or AIDS dementia. In a series of 677 AIDS patients, 27% had bladder hyperreflexia.[22,23] Treatment of hyperreflexia includes anticholinergic therapy. More often, bladder hyporeflexia or areflexia (45%) are encountered.[22,23] Areflexia is a result of lower motor neuron injury, usually caused by malignancy or infection (herpes). This results in urinary retention, stasis, and urinary tract infections.[43] Areflexia is best treated with clean intermittent catheterization (CIC), but can also be treated with foley catheterization or suprapubic cystostomy if CIC is impossible. It is essential that patients with urgency, incontinence, or retention be evaluated by the urologist. Urine cultures are mandatory, and a cystogram should be performed in symptomatic patients.

**TABLE 1**
**Diagnostic Modalities and Therapeutic Regimens in Ten Patients with Prostatic Abscesses.**

| Patient | Age | Method of Diagnosis | Initial Culture | Operative Culture | Surgical Treatment | Medical Therapy |
|---|---|---|---|---|---|---|
| 1 | 33 | DRE | Negative | No growth | TUR unroofing | IV antibiotics |
| 2 | 43 | CT | Negative | MAI | Perineal drainage/ SPT | Antimycobacterial |
| 3 | 50 | TRUS | Negative | No surgery | Refused | Oral ciprofloxacin |
| 4 | 35 | CT | *S. aureus* | No surgery | No Surgery | Long-term IV vancomycin |
| 5 | 33 | TRUS | Negative | No growth | TUR unroofing | Long-term ciprofloxacin |
| 6 | 42 | TRUS | Negative | *M. tuberculosis* | TUR unroofing | Antimycobacterial |
| 7 | 34 | TRUS | Negative | *Enterococcus* | TUR unroofing/SPT | Long-term amoxicillin |
| 8 | 33 | CT | Negative | No growth | TUR unroofing | No growth |
| 9 | 43 | CT | Negative | *Aspergillus* | Diagnostic aspiration | IV amphotericin |
| 10 | 68 | CT | Negative | *Enterococcus* | Perineal, transrectal drainage/SPT | Long-term ciprofloxacin |

KEY: DRE = digital rectal exam, CT = computerized tomography, TRUS = transrectal ultrasound, MAI = *mycobacterium avium intracellulare*, TUR = transurethral resection, SPT = suprapubic tube

Adapted from Trauzzi et al[32], Staiman and Lowe[33], and Kay et al[34]

### TESTICULAR ATROPHY

Atrophy of the testicles is usually related to advanced age, alcohol/cirrhosis, and cigarette smoking. In patients with AIDS, atrophy is the most prevalent AIDS-associated testicular disorder and is related to chronic illness, prolonged fever, malnutrition and cachexia.[44-46]

The histopathologic changes found in the HIV-positive patient population that are not often found in the general population include peritubular interstitial inflammation and interstitial fibrosis.[47] Vascular changes, including smaller vessel lumen size and intravascular thrombosis, lead to ischemia and ultimate atrophy.[44] Additionally, spermatogenesis is decreased and maturation arrest can occur. [44,46] Twenty-eight to 38% of all male patients with AIDS have an abnormal hypothalamic-pituitary-testicular axis causing atrophy.

HIV itself can primarily cause atrophy by having cytotoxic effect on germinal tissue and on Sertoli cells. HIV is also associated with secondary causes of testicular atrophy. CMV is the most common infective agent of the testes, and 26% of HIV-infected patients with systemic CMV have testicular involvement.[44] *Toxoplasma* and *Mycobacterium* have also been reported.

Many HIV-positive/AIDS patients are being treated, often long-term, with a variety of antibiotics and antifungal agents. In addition, fifteen percent of HIV-positive and 30 to 40% of AIDS patients will also develop carcinoma during their lifetime.[17,48] and many will receive chemotherapy. These medications are known to be gonadotoxic, producing atrophy.[44]

### ERECTILE DYSFUNCTION

Erectile and ejaculatory dysfunction also occurs in HIV-infected patients. Erectile dysfunction is often caused by psychologic and neurogenic factors. Patients with AIDS may suffer from fatigue and depression; this leading to decreased libido. Neurogenic factors include infections (viral myelitis/myelopathies), malignancy, and AIDS dementia. Dobs and associates reported 33% impotence and 66% decreased libido.[49] In another study by Tindall and colleagues, difficulty maintaining erections and achieving ejaculation was the most frequently noted dysfunction in AIDS patients. Seventeen percent could not obtain an erection or ejaculate at all.[50] Many patients with AIDS also have hypogonadism and low testosterone levels[2] and this may cause decreased libido and erectile dysfunction. Testosterone patches and injections, which are currently being used to prevent cachexia, have been correlated with a decreased incidence of impotence.[13]

### NEPHROPATHY

HIV disease is associated with a variety of renal syndromes. Mild to moderate proteinuria occurs in 38 to 82% of HIV-seropositive patients while nephrotic-like proteinuria is seen in approximately 10%.[16,51,52] Fluid and electrolyte changes, including hyponatremia in 12 to 30% (from volume depletion)[53] and hypokalemia (from diarrhea, vomiting) are seen and often worsen with progression of HIV disease.[53-55] Prompt attention to electrolyte abnormalities may prevent the development of acute tubular necrosis and subsequent renal failure.[53]

HIV associated nephropathy (HIVAN), first reported in 1984, is seen in 5 to 10% of HIV-infected patients[56,57] and is most commonly seen in black males with a history of intravenous drug abuse.[52,58,59] It is not usually associated with hypertension. Histopathologic features include diffuse, global sclerosis, epithelial cell hypertrophy, severe tubulo-interstitial inflammation, edema and dilatation of tubules.[52,60] On ultrasound, large, echogenic kidneys are seen, and pelvocalyceal thickening can occur.[54,61] There is usually progression to end stage renal disease (ESRD) within 3 to 6 months.[62-64] Patients can be treated by either peritoneal or hemodialysis[65], but hemodialysis does not seem to prolong life in patients with AIDS[66] as there is a 95% mortality rate within 6 months.[67]

Preliminary studies suggest that treatment of HIVAN with Zidovudine, a thymidine analogue which decreases HIV replication, may result in clinical improvement of HIVAN[60,68], and that discontinuations may cause an irreversible, rapid loss of renal function[68].

Sepsis in HIV patients will often cause acute tubular necrosis (ATN) and subsequent acute renal failure (ARF). ARF is seen in 6 to 20% of AIDS patients who are hospitalized and the mortality rate approaches 70% in hemodynamically unstable AIDS patients.[53] In addition, numerous medications taken by HIV-positive patients are nephrotoxic and are responsible for ATN and ARF. Trimethoprim-sulfamethoxazole, which is used as prophylaxis for *P. carinii*, causes elevation is serum creatinine and ATN.[18] Foscarnet, currently used in the treatment of CMV, causes renal alterations which can be reversible. Other nephrotoxic drugs include cidofovir, and acyclovir.[18]

### NEPHROLITHIASIS

### Indinavir

Indinavir (Crixivan) is commonly used in the treatment regimen for patients with AIDS. Indinavir acts as a protease inhibitor. This inhibitor competitively binds to the active site of the HIV protease ultimately preventing the formation of new viral particles.[69,70] The usual dosage of indinavir is 800 mg three times per day. Approximately 80% of the drug is metabolized in the liver and is eliminated in the feces, while the other 20% is not metabolized and is excreted by the kidneys within 24 hours.[69-72] The solubility of the non-metabolized drug in water is pH-dependent, and is most soluble at pH less than 5.[70] Since the normal urinary pH is usually greater than 5, the drug is found to precipitate in the urine as indinavir crystals.

Nephrolithiasis has been recently studied in HIV/AIDS patients taking indinaivir. 2.6 to 4% of patients on the recommended dose of 2.4 gm/day have symptoms of nephrolithiasis.[18,69,70,73,74] Most patients have flank pain which is often, but not always, associated with microscopic hematuria.[18,69,73] Indinavir calculi are radiolucent; however, indinavir may act as a nidus for the formation of mixed-type stones.[71,72] Calcium oxalate and calcium phosphate may also be present as part of the make-up of these stones, and thus the calculus may be partly radioopaque.[72] Since indinavir has been found at the central area of mixed-calculi, it is believed that indinavir may be lithogenic, especially in those patients with underlying metabolic abnormalities.[71,72] Intravenous pyelogram, renal ultrasound, or non-contrast spiral CT scan may be helpful in the diagnosis. In one study which summarized the patient characteristics while taking this drug, 7% of the patients taking higher doses of indinavir had symptoms of nephrolithiasis.[73] Nine percent of the patients stopped using indinavir after the episode of nephrolithiasis. Of those who continued on their medication regimen, 8% had stone formation recurrence. Kopp and colleagues recently reported on 240 patient receiving indinavir as part of their therapeutic regimen compared to a 40 patient control arm.[74] Twenty percent of patients had urinary indinavir crystals confirmed by mass spectrometry and high-performance liquid chromatography. They noted that 8% of patients receiving indinavir had urinary symptoms with 3% actually having nephrolithiasis. Five percent also had crystalluria with either dysuria or flank pain. One third of these actually had intrarenal sludging visualized radiographically. None of the patients in the control arm had crystalluria or nephrolithiasis.

Initial reports suggest that patients with indinavir calculi may be treated conservatively with hydration, analgesia, and a 1 to 3 day interruption in indinavir ingestion.[73,75] Daudon et al report that 83% had stone passage with conservative therapy while only 17% needed stone extraction.[72] The most recent report by Bruce et al; however, suggests that surgical intervention is often required to relieve both obstruction and pain.[71]

In a study of 11 patients at our institution taking the recommended dosage of indinavir, all 11 patients required temporary stenting, usually external, for relief of obstruction and pain within 12 to 48 hours.

In order to help prevent stone formation in patients taking indinavir, increased fluid intake is essential. The pharmakokinetics of this medication indicate that with a daily urine output of 1500 cc, the urinary concentration of indinavir is 0.2 to 0.3 mg/ml. This is the limit of solubility of indinavir at a urinary pH of 5. At higher pH, the solubility is even less. The normal urinary pH is often between 5.5 and 8; however, it is difficult to maintain a low urinary pH such as 5. Low urinary pH is also associated with uric acid stone formation, and in the case of indinavir, may also result in hypocitraturia and calcium oxalate supersaturation.[71] An increased urine output would decrease the concentration of indinavir in the urine, making it soluble at a higher pH.[72]

For patients with indinavir stones/sludging causing high-grade obstruction, it is our recommendation that these patients should be treated with temporary stenting. Often at the time the procedure, an actual stone is not visualized but rather a large amount of sediment is present in the ureter. In these patients, an open-ended ureteral catheter may be placed for 1 to 2 days until the patient is afebrile. This is subsequently removed at the bedside; therefore, not warranting another procedure. Patients with a history of calculi are strongly suggested to increase fluid consumption as this may decrease their risk of both indinavir, and mixed-type calculi. Patients are not required to discontinue their treatment regimen, but are warned of the future risk of stone formation while on this medication.

### NEOPLASMS

### Kaposi's Sarcoma

Kaposi's sarcoma (KS), a disease of the reticulendothelial system, has been the most common malignancy associated with AIDS.[76,77] Recently, there appears to be a decreasing incidence of these lesions. Previously approximately 4% of patients diagnosed with AIDS will have KS as their presenting symptom[1], 30% will develop it during their illness, and approximately 20% will eventually develop genital lesions.[78] It appears that a herpes virsus (KSHV) or CMV may be related to the development of KS in this population.[79-81]

Clinically, KS appears as a purple, papular, plaque-like, or ulcerated lesion found on the penis or scrotum. Other areas of cutaneous involvement and/or systemic involvement are also noted. The kidney is a rare site, as less than 10 cases have been reported in the literature.[82] All cases were of homosexual males who had cutaneous and multifocal visceral involvement.

Presentation with KS of the penis as a primary presentation of AIDS usually represents widespread disease. KS can cause pain and edema, as well as irritative and obstructive voiding symptoms.[17] Urinary retention is possible[76], especially with lesions of the urethral meatus[83], and urinary diversion with suprapubic cystostomy may be required until treatment is planned. Due to the arteritis and arterial occlusion caused by this disease, KS of the penis can eventually progress to gangrene.[84]

Treatment for KS is based on the size of the lesion, its location, and the evidence of systemic involvement.[78] Small lesions of the penis can be locally excised, removed with laser therapy or radiated[78,85], while larger lesions are best treated with radiation therapy. Early, vascular KS lesions appear to be treated more effectively with radiation therapy than lesions that are older and indurated.[86] Disseminated KS is treated with chemotherapy; doxorubicin, bleomycin, and vincristine, with a total response rate of 88%, seems to have the best efficacy with the least toxicity. [84,87] Alpha-interferon has also had varied success (20 to 50%) but is not as efficacious in patients with CD4 counts of less than 600.[84,87] Recently, the protease inhibitor, ritonavir, was found to cause improvement or resolution of KS lesions in 5/5 patients.[88] The mechanism of action is not clear at this time, but it has been proposed by the investigators that a decreased viral load improves immunologic function and this negatively influences KS growth. Further investigation with large, controlled studies is required.

### Testicular Malignancy

Testicular neoplasms are the third most common AIDS-associated malignancy, following KS and non-Hodgkins lymphoma.[44] The incidence of testicular malignancy in the non-HIV populations is 0.004% while it is 0.2% in those with HIV (especially black and hispanic patients).[44,89,90] The usual association with the formation of testicular malignancies (cryptorchidism) is not seen in this population. While seminomatous-type tumors are most common in the general population[89], non-seminomatous tumors are more common in HIV-positive patients.[76,89,91-93] Fortunately, most tumors are either stage I or II, and respond equally as well as those found in non-HIV-infected patients.

Seminomatous tumors respond well to radiation therapy[94], while non-seminomatous tumors are usually treated with chemotherapy. Treatment in this population is controversial. Some suggest surveillance for all stage I tumors in AIDS patients because treatment can further potentiate immunosuppression.[93] Conversely, asymptomatic HIV-positive patients should be offered all treatment options as most will tolerate therapy well. [95] Tumor relapse has been reported with decreasing immunologic status[89], but most patients will die from HIV related complications before relapse can occur.[44]

### Lymphoma

Lymphoma accounts for 5% of testicular malignancies[96] and occurs with increasing frequency in patients older than 50 years.[97] If a primary B-cell lymphoma is found in a person younger than 50, it implies compromised immunologic status, most likely, AIDS.[98] The most common presenting symptom is testicular enlargement[97] and bilateral testicular masses also suggest the diagnosis. Histopathologically, diffuse histiocytic lymphoma is seen.[96] At the time of diagnosis, the disease is often widely metastatic. Lymphoma also affects other parts of the genitourinary system including the kidneys (6 to 12%)[18], and ureters. Urinary obstruction can occur, causing anuric renal failure, which may require ureteral stenting or percutaneous nephrostomy placement.[18,96]

Most lymphomas can be treated with radiation therapy, with or without adjuvant chemotherapy. These tumors are responsive to chemotherapeutic agents with a 2 year disease-free survival being 50 to 80%.[96] After temporary remission; however, a rapid course of the disease transpires.

### CONCLUSION

HIV both primarily and secondarily affects virtually every part of the genitourinary system. As patients with HIV/AIDS are living longer lives due to markedly improved therapy, it is important for the urologist to be familiar with these manifestations, and the treatment options available.

### REFERENCES

1. AIDS surveillance-general epidemiology. www.cdc.gov /nchstp /hiv_aids /graphics / surveill.htm.
2. Levy, J.A.: The transmission of HIV and factors influencing progression to AIDS. Am J Med 95: 86-100, 1993.
3. Critchlow, C.W., Kiviat, N.B.: Detection of human immunodeficiency virus type 1 and type 2 in the female genital tract: implications for the understanding of virus transmission. Obstet Gynecol Surv 52: 315-325, 1997.
4. European Study Group on Heterosexual transmission of HIV: comparison of female to male and male to female transmission of HIV in 563 stable couples. Br Med J 304: 809-813, 1992.
5. Fenyo, E.M., Fiore, J., Karlsson, A., et al: Biologic phenotypes of HIV-1 in pathogenesis and transmission. Antibiot Chemother 46: 18-24, 1994.
6. Kar, P.K.: A study on acquired immune deficiency syndrome: transmission and diagnosis-a review. J Indian Med Assoc 94: 143-144, 1996.
7. Royce, R.A., Sena, A., Cates, W., et al: Sexual transmission of HIV. N Engl J Med 336: 1072-1078, 1997.
8. Alexander, N.J.: Sexual transmission of human immunodeficiency virus: virus entry into the male and female genital tract. Fertil Steril 54: 1-18, 1990.
9. Moses, S., Plummer, F.A., Bradley, J.E., et al: The association between lack of male circumcision and risk for HIV infection: a review of the epidemiological data. STD 21: 201-210, 1994.
10. Cameron, D.W., Simonsen, J.N., D'Costa, L.J., et al: Female to male transmission of human immunodeficiency virus type 1: risk factors for seroconversion in men. Lancet 2: 403-407, 1989.
11. Kreiss, J.K., Hopkins, S.G.: The association between circumcision status and human immunodeficiency virus infection among homosexual men. J Infect Dis 168: 1404-1408, 1993.
12. Marx, J.L.: Circumcision may protect against the AIDS virus. Science 245: 470-471, 1989.
13. Kwan, D.J., Lowe, F.C.: AIDS and the urologist. Part I (lesson 37 ). AUA Update Series 14: 297-304, 1995.
14. Vernazza, P.L., Gilliam, B.L., Flepp, M., et al: Effect of antiviral treatment on the shedding of HIV-1 in semen. AIDS 11: 1249-1254, 1997.
15. Miles, B.J., Mesler, M., Farah, R., et al: The urological manifestations of the acquired immunodeficiency syndrome. J Urol 142: 771-773, 1989.
16. Kaplan, M.S., Wechsler, M., Benson, M.C.: Urologic manifestations of AIDS. Urology 30: 441-443, 1987.
17. Vapneck, J.M.: Urologic disease in HIV infection. The AIDS Reader 1: 64-72, 1991.
18. Steele, B.W., Carson, C.C.: Recognizing the urologic manifestations of HIV and AIDS: Contemp Urol 9: 39-53, 1997.
19. De Pinho, A.M., Lopes, G.S., Ramos-Filho, C.F., et al: Urinary tract infections in men with AIDS. Genitourin Med 70: 30-34, 1994.
20. Hoepelman, A.I., Van Buren, M., Van den Broek, J., et al: Bacteriuria in men infected with HIV-1 is related to their immune status (CD4+ cell count). AIDS 6: 179-184, 1992.
21. Evans, J.K., McOwan, A., Hilman, R.J., et al: Incidence of symptomatic urinary tract infections in HIV seropositive patients and the use of cotrimazole as prophylaxis against pneumocystis carinii pneumonia. Genitourin Med 71: 120-122, 1995.
22. Catanese, A.J., Tessler, A.N., Morales, P.: AIDS and the urologist part I: urologic manifestation of AIDS (lesson 1). AUA Update Series 8: 1-8, 1989.
23. Kahn, Z., Singh, V.K.: Neurogenic bladder in acquired immune deficiency syndrome. J Urol 139: 166A, 1988 (abstract).
24. Cespades, R.D., Peretsman, S.J., Blat, S.P.: The significance of hematuria in patients infected with the human immunodeficiency virus. J Urol 154: 1455-1456, 1995.
25. Weinberger, M., Cytron, S., Servadio, C., et al: Prostatic abscess in the antibiotic era. Rev Infect Dis 10: 239-249, 1988.
26. Fair, W.R., Couch, J., Wehner, N.: The purification and assay of the prostatic antibacterial factor (PAF). Biochem Med 8: 329-339, 1973.
27. Leport, C., Rousseau, F., Perronne, C., et al: Bacterial prostatitis in patients infected with the human immunodeficiency virus. J Urol 141: 334-336, 1989.
28. Trapnell, J., Roberts, M.: Prostatic abscess. Br J Surg 57: 565-569, 1970.
29. Benson, P.J., Smith, C.S.: Cytomegalovirus prostatitis. Urology 40: 165-167, 1992.
30. Larsen, R.A., Bozzette, S., McCutchan, J.A., et al: Persistent cryptococcus neoformans infection of the prostate after successful treatment of meningitis. Ann Intern Med 11: 125-128, 1989.
31. Centers for Disease Control and Prevention: HIV/AIDS surveillance report 6(1): 5-14, 1994.
32. Trauzzi, S.J., Kay, C.J., Kaufman, D.G., et al: Management of prostatic abscess in patients with the human immunodeficiency virus syndrome. Infect in Urol 8: 86-90, 1995.
33. Staiman, V.S., Lowe, F.C.: Prostatic disease in HIV-infected patients. The AIDS Reader 5: 165-171, 1995.
34. Kay, C.J., Trauzzi, S.J., Lowe, F.C.: Imaging and management of prostatic abscess associated with the human immunodeficiency virus syndrome. Infect in Urol 8: 86-90, 1995.
35. Mamo, G.J., Rivero, M.A., Jacobs, S.C.: Cryptococcal prostatic abscess associated with the acquired immunodeficiency syndrome. J Urol 148: 889-890, 1992.
36. Marans, H.Y., Mandell, W., Kislak, J.W., et al: Prostatic abscess due to histoplasma capsulatum in the acquired immunodeficiency syndrome. J Urol 145: 1275-1276, 1991.
37. Zighelboim, J., Goldfarb, R.A., Mody, D., et al: Prostatic abscess due to histoplasma capsulatum in a patient with acquired immunodeficiency syndrome. J Urol 147: 166-168, 1992.
38. Adams, J.R., Mata, J.A., Culkin, D.J., et al: Acquired immunodeficiency syndrome manifesting as a prostate nodule secondary to cryptococcal infection. Urology 29: 289-291, 1992.
39. Lief, M., Sarfarazi, F.: Prostatic cryptococcus in acquired immune deficiency syndrome. Urology 28: 318-319, 1986.
40. Gyrtrup, H.J., Kristiansen, V.B., Zachariae, C.O., et al: Voiding problems in patients with HIV infection and AIDS. Scand J Urol Nephrol 29: 295-298, 1995.
41. Menendez, V., Espuna, M., Perez, A., et al: Neurogenic bladder in patients with acquired immunodeficiency syndrome. Neurourology and Urodynamics 14: 253-257, 1995.
42. Hermieu, J.F., Delma, V., Boccon-Gibod, L.: Micturition disturbances and human immunodeficiency virus infection. J Urol 156: 157-159, 1996.
43. Kwan, D.J., Lowe, F.C.: Acquired immunodeficiency syndrome, a venereal disease. Urol Clin North Am 19: 13-24, 1992.
44. Leibovitch, I., Goldwasser, B.: The spectrum of acquired immunodeficiency syndrome-associated testicular disorders. Urology 44: 818-824, 1994.
45. Pudney, J., Anderson, D.: Orchitis and human immunodeficiency virus type I infected cells in reproductive tissues from men with the acquired immune deficiency syndrome. Am J Pathol 139: 149-160, 1991.
46. DePaepe, M.E., Waxman, M.: Testicular atrophy in AIDS: a study of 57 autopsy cases. Human Pathol 20: 210-214, 1989.
47. DePaepe, M.E., Guerrieri, C., Waxman, M.: Opportunistic infections of the testis in the acquired immunodeficiency syndrome. Mt Sinai J Med 57: 25-29, 1990.
48. Adjiman, S., Zerbib, M., Flam, T., et al: Genitourinary tumors and HIV-1 infection. Eur Urol 18: 61-63, 1990.
49. Dobs, A.S., Dempsey, M.A., Landenson, P.W., et al: Endocrine disorders in men infected with human immunodeficiency virus. Am J Med 84: 611-614, 1988.
50. Tindall, B., Forde, S., Goldstein, D., et al: Sexual dysfunction in advanced HIV disease. AIDS Care 6: 105-107, 1994.
51. Benson, M.C., Kaplan, M.S., O’Toole, K., et al: A report of cytomegalovirus cystitis and a review of other genitourinary manifestations of the acquired immune deficiency syndrome. J Urol 140: 153-154, 1988.
52. Obayomi, O.O., Dillard, M.G., Hosten, A.O., et al: End-stage renal disease in patients infected with human immunodeficiency virus: a retrospective of 38 cases. J Natl Med Assoc 87: 762-765, 1995.
53. Rao, T.K.S.: Renal complications in HIV disease. Med Clin North Am 80: 1437-1451, 1996.
54. Glassock, R.J.: Human immunodeficiency (HIV) infection and the kidney. Ann Intern Med 112: 35-49, 1990.
55. Bourgiognie, J.J., Pardo, V.: The nephropathology in human immunodeficiency virus (HIV-1) infection. Kidney Int 35: S19-S23, 1991.
56. Rao, T.K., Filippone, E.J., Nicastri, A.D., et al: Associated focal and segmental glomerulosclerosis in acquired immunodeficiency syndrome. N Eng J Med 310: 669-673, 1984.
57. Rao, T.K.S., Friedman, E.A., Nicastri, A.D.: The types of renal disease in the acquired immunodeficiency syndrome. N

Eng J Med 316: 1062-1068, 1987.
58. Noch, D., Glotz, D., Dosquet, P., et al: Renal disease associated with HIV infection: a multicentre study of 60 patients from Paris hospitals. Nephrol Dial Transplant 8: 11-19, 1993.
59. Weening, J.J., Beukers, J.J., Grond, J., et al: Genetic factors in focal segmental glomerulosclerosis. Kidney Int 29: 789-798, 1986.
60. Stone, H.D., Appel, R.G.: Human immunodeficiency virus-associated nephropathy: current concepts. Am J Med Sci 307: 212-217, 1994.
61. Wachsberg, R.H., Obolevich, A.T., Lasker, N.: Pelvocalyceal thickening in HIV-associated nephropathy. Abdom Imaging 20: 371-375, 1995.
62. Mazbar, S.A., Schoenfeld, P.Y., Humphreys, M.H.: Renal involvement in patients infected with HIV: experience at San Francisco general hospital. Kidney Int 37: 1325-1332, 1990.
63. Rao, T.K.S.: Clinical features of human immunodeficiency virus associated nephropathy. Kidney Int 40: S13-S18, 1991.
64. Frassetto, L., Schoenfeld, P.Y., Humphreys, M.H.: Increasing incidence of human immunodeficiency virus associated nephropathy at San Francisco general hospital. Am J Kidney Dis 18: 655-659, 1991.
65. Coleburn, N.H., Scholes, J., Lowe, F.C.: Renal failure in patients with AIDS-related complex. Urology 37: 523-527, 1991.
66. Ismail, N., Becker, B.N.: Treatment options and strategies in uremia: current trends and future directions. Sem in Nephrol 14: 282-299, 1994.
67. Vapneck, J.M., Mc Aninch, J.W.: AIDS and the urologist. Infect Urol 4: 101-107, 1990.
68. Ifudu, O., Rao, T.K.S., Tan, C.C., et al: Zidovudine is beneficial in human immunodeficiency virus associated nephropathy. Am J Nephrol 15: 217-221, 1995.
69. Heylen, R., Miller, R.: Adverse effects and drug interactions of medications commonly used in the treatment of adult HIV positive patients: part 2. Genitourin Med 73: 5-11, 1997.
70. Tashima, K.T., Horowitz, J.D., Rosen, S.: Indinavir nephropathy. New Engl J Med 336: 138-139, 1997.
71. Bruce, R.G., Munch, L.C., Hoven, A.D., et al: Urolithiasis associated with the protease inhibitor indinavir. Urology 50: 513-518, 1997.
72. Daudon, M., Estepa, L., Viard, J.P., et al: Urinary stones in HIV-1 positive patients treated with indinavir. Lancet 349: 1294-1295, 1997.
73. Crixivan (indinavir sulfate)-US package insert. West Point, PA: Merck and Co., 1996.
74. Kopp, J.B., Miller, K.D., Mican, J.M., et al: Crystalluria and urinary tract abnormalities associated with indinavir. Ann Intern Med 127: 119-125, 1997.
75. Sutherland, S.E., Reigle, M.D., Seftel, A.D., et al: Protease inhibitors and urolithiasis. J Urol 158: 31-33, 1997.
76. Kennedy, W.A., Benson, M.C., Kaplan, S.A.: Urologic Manifestations of HIV Infection: Renal and Urologic Aspects of HIV Infection. New York: Churchill Livingstone, pp. 181-193, 1995.
77. Piedbois, P., Frikha, H., Martin, L., et al: Radiotherapy in the management of epidemic Kaposi's sarcoma. Int J Radiation Oncology Biol Phys 30: 1207-1211, 1994.
78. Lowe, F.C., Lattimer, G.D., Metroka, C.E.: Kaposi's sarcoma of the penis in patients with acquired immunodeficiency syndrome. J Urol 136: 673-675, 1986.
79. Chang, Y., Cesarman, E., Pessin, M., et al: Identification of herpesirus-like DNA sequence in AIDS-associated Kaposi's sarcoma. Science 266: 1865-1869, 1994.
80. Foreman, K.E., Friborg, J., Kong, W., et al: Propagation of a human herpesvirus from AIDS-associated Kaposi's sarcoma. N Engl J Med 336: 163-171, 1997.
81. Dejarlais, D.C.: Kaposi's sarcoma amongst four different AIDS risk groups. N Engl J Med 310: 1119, 1984.
82. Pollok, R.C.G., Francis, N., Cliff, S., et al: Kaposi's sarcoma in the kidney. Intl J STD AIDS 6: 289-290, 1995.
83. Swierzewski, S.J. 3d, Wan, J., Boffini, A., et al: The management of meatal obstruction due to Kaposi's sarcoma of the glans penis. J Urol 150: 193-195, 1993.
84. Klein, L.T., Lowe, F.C.: Penile gangrene associated with extensive Kaposi's sarcoma in patients with the acquired immunodeficiency syndrome. Urology 46: 425-428, 1995.
85. Kwan, D.J., Lowe, F.C.: Acquired immunodeficiency syndrome in urology. part II: genitourinary manifestations of AIDS (lesson 38), AUA Update Series 14: 313-320, 1995.
86. Nisce, L.Z., Safai, B., Poussin-Rosillo, H.: Once weekly total and subtotal skin electron beam therapy for Kaposi's sarcoma. JAMA 252: 934, 1984.
87. Safai, B., and Schwartz, J.J.: Kaposi's sarcoma and the acquired immunodeficiency syndrome, in Devita VT Jr. Hellman S, and Rosenberg SA Eds): AIDS: Etiology, Diagnosis, Treatment and Prevention, 3rd ed. Philadelphia: JB Lippincott, pp. 209-223, 1992.
88. Conant, M.A., Opp, R.M., Foretz, D., et al: Reduction of Kaposi's sarcoma lesions following treatment of AIDS with ritonovir. AIDS 11: 1300-1301, 1997.
89. Wilson, T.W., Frenkel, E., Vultch, F., et al: Testicular tumors in men with human immunodeficiency virus. J Urol 147: 1038-1040, 1992
90. Moyle, G., Hawkins, D.A., Gazzard, B.G.: Seminoma and HIV infections. Int J STD AIDS 2: 293-294, 1991.
91. Tessler, A.N., Catanese, A.: AIDS and germ cell tumors of testis. Urology 30: 203-204, 1987.
92. Kaplan, M.H., Susin, M., Pahwa, S.G., et al: Neoplastic complications of HTLV-III infection: lymphomas and solid tumors. Am J Med 82: 389-396, 1987.

93. Wilkinson, M., Carroll, P.R.: Testicular carcinoma in patients positive and at risk for human immunodeficiency virus. J Urol 144: 1157-1159, 1990.
94. Monfardini, S., Vaccher, E., Pizzocaro, G., et al: Unusual malignant tumors in 49 patients with HIV infection. AIDS 3: 449-452, 1989.
95. Leibovitch, I., Baniel, J., Rowland, R.G., et al: Malignant testicular neoplasms in immunosuppressed patients. J Urol 155: 19938-1942, 1996.
96. Mohler, J.L., Jarow, J.P., Marshall, F.F.: Unusual urological presentations of acquired immune deficiency syndrome: large cell lymphoma. J Urol 138: 627-629, 1987.
97. Sokovich, R.S., Bormes, T.P.: Acquired immunodeficiency syndrome presenting as testicular lymphoma. J Urol 147: 1110-1111, 1992.
98. Armenakas, N.A., Schevchuk, M.M., Brodherson, M, et al: AIDS presenting as primary testicular lymphoma. Urology 40: 162-164, 1992.

**This article should be referenced as follows:**

Staiman, V.R. and Lowe, F.C. (2004) Urologic problems in patients with acquired immunodeficiency syndrome. *TheScientificWorldJOURNAL* **4 (S1)**, 427–437.

**Handling Editor:**

Anthony Atala, Principle Editor for *Urology* — a domain of *TheScientificWorldJOURNAL*.
